# Effects of In Ovo Injection of Mannan-Oligosaccharides on Early Growth Performance, Intestinal Morphology, and Cecal Microbiota in Broilers

**DOI:** 10.3390/ani16172802

**Published:** 2026-09-06

**Authors:** Chentong Wang, Manyang Jin, Yufan Li, Yilong Lv, Ju Chai, Haitao Shi, Xue Bai, Yanling Huang, Xi Wang

**Affiliations:** College of Animal and Veterinary Sciences, Southwest Minzu University, Chengdu 610041, China; 202330903058@stu.swun.edu.cn (C.W.); 202330903069@stu.swun.edu.cn (M.J.); 202430903029@stu.swun.edu.cn (Y.L.); 240905022007@stu.swun.edu.cn (Y.L.); 240905022002@stu.swun.edu.cn (J.C.); shihaitao@swun.edu.cn (H.S.); baixue@swun.edu.cn (X.B.); swunylh@163.com (Y.H.)

**Keywords:** mannan-oligosaccharides, in ovo injection, prebiotics, broiler chickens, cecal microbiota, intestinal morphology

## Abstract

Mannan oligosaccharides (MOS) are functional prebiotics that selectively promote the proliferation of beneficial bacteria in the chicken intestinal tract. This study evaluated the effects of in ovo MOS injection on early growth, intestinal morphology, and cecal microbiota of broilers at 7, 14, and 21 days post-hatch (dph). In total, 700 Cobb 500 fertilized eggs received either no injection or 200 μL of PBS diluent, low (0.5 mg/egg), medium (5 mg/egg), or high (10 mg/egg) doses of MOS in the amnion. At hatch, approximately 280 male hatchlings from injection groups were distributed into 28 cages and raised until 21 dph. Our results indicated that MOS injection linearly increased feed intake and body weight during 0–7 dph and improved duodenal villus height and width, with no persistent improvements observed at older age. Moreover, microbiota shifts triggered by MOS were only observed at 7 dph. The MOS injection at 0.5 mg/egg reduced genus-level Sobs, and PCoA coupled with ADONIS analysis confirmed microbial separation across treatments (*p* = 0.017). MOS selectively enriched obligate anaerobic taxa, including Lachnospiraceae, Pseudoflavonifractor, and Collidextribactor (0.5 mg/egg), and Ruminococcus gauvreauii (5 mg/egg). Conversely, control-enriched genera (Enterococcus and Litchfieldia) were negatively correlated with body weight gain. In conclusion, in ovo administration of MOS at 0.5 mg/chick transiently improved the abundance of beneficial anaerobes, and injecting 10 mg MOS could improve villus morphology and growth performance in chicks in the first week, suggesting a potential role for MOS in shaping early microbial establishment in broilers.

## Simple Summary

This study explored the effects of in ovo injection of mannan oligosaccharide (MOS), a functional prebiotic fiber, on broiler early growth, intestinal structure, and gut microbiota. A total of 700 Cobb 500 fertilized eggs received either no injection or in ovo injection of PBS diluent or low (0.5 mg/egg), medium (5 mg/egg), and high (10 mg/egg) doses of MOS at embryonic day 17.5. After hatching, male chicks from injected groups were raised for 21 days to evaluate early growth and gut health. The results showed that MOS injection linearly improved feed intake and body weight of chicks during days 0–7. In addition, MOS enhanced intestinal villus structure and modulated microbiota diversity and composition in 7-day-old broilers, with no significant impacts on older chicks. MOS selectively enriched obligate anaerobic taxa, including Lachnospiraceae, Pseudoflavonifractor, Collidextribactor, and Ruminococcus gauvreauii. Overall, in ovo MOS supplementation may promote intestinal structure while modulating gut microbiota in young broilers, with effects that appear to be transient during the first week post-hatch.

## 1. Introduction

The establishment of intestinal microbiota in neonatal chicks is a programmed successional process, typically beginning within hours at hatch [1]. Initially dominated by facultative anaerobes such as Enterobacteriaceae, the community rapidly transitions toward obligate anaerobes like Bacteroidetes and Firmicutes [2], achieving a stable diversity around 21–28 days post-hatch (dph) in broilers [3]. Gut microbiota shapes within a “critical window of opportunity,” during which early-life interventions exert profound and long-lasting effects on host health [4]. In ovo injection, originally performed to deliver vaccines, allows exogenous nutritive compounds to enter the digestive tract prematurely prior to hatching and to intervene in microbiota colonization sooner [1]. This early programmed microbiota can prevent pathogen invasion, facilitate bird innate immunity, and influence the life-term growth performance of broilers [1,5].

Prebiotics are non-digestible carbohydrates that benefit the host by promoting growth and activity of selective microbes, potentially producing short-chain fatty acids (SCFAs) and other metabolites that contribute to intestinal homeostasis in the host [6]. Mannan oligosaccharides (MOS), oligomers of mannose linked by β-1,4 glycosidic bonds, are utilized as prebiotics due to their proven ability to inhibit intestinal pathogens and bolster the immune system [7,8]. MOS binds to specific receptors on the surface of pathogens like *Salmonella* and *Escherichia coli*, exerting a “flushing” effect that blocks their adhesion to the intestinal mucosa [9,10,11]. Concurrently, MOS supplementation elevates immunoglobulins (IgA, IgG, and IgM), strengthening systemic humoral defense [12]. In ovo administration of MOS during late incubation accelerates intestinal barrier maturation and primes the innate immune system before microbial colonization [13]. Previous studies have demonstrated that dietary inclusion of MOS increases body weight and improves feed efficiency in broilers [14]. Furthermore, MOS intervention promotes mucosal structural development, increasing villus height and improving the villus height-to-crypt depth ratio in the small intestine [15]. Previous studies have demonstrated that amniotic injection of nutrients and prebiotics enhances immune competence, modulates gut microbiota, and promotes post-hatch growth and development in chicks [5].

While the first week of a chick’s life is widely recognized as a “window of opportunity” characterized by high microbiome plasticity, capturing this window even earlier, during the pre-hatch embryonic stage, has become a strategic focus in poultry production [16,17]. Importantly, interventions during this early developmental period may not only alter the abundance of individual bacterial taxa [18] but also influence the trajectory of microbial succession as the intestinal ecosystem matures [19]. However, whether microbiota changes induced by in ovo prebiotic administration persist beyond the immediate post-hatch period remains unclear. The present study administered MOS during the late stages of embryonic development to modulate intestinal microbiota and also investigate its impacts on the growth performance and intestinal health of broilers in early life.

## 2. Materials and Methods

### 2.1. Experimental Design and Bird Management

The present study was conducted in an environmentally controlled poultry facility at Southwest Minzu University. The chick and egg handling procedures and experimental protocols were approved by the Academic Ethics Committee at Southwest Minzu University (SMU-202409007).

A randomized complete block design was employed to evaluate the effects of in ovo MOS administration on growth performance, intestinal morphology, and cecal microbiota in broilers from 0 to 21 dph. A total of 700 Cobb 500 fertilized eggs were randomly allocated into seven tiers (served as blocks) in an automatic incubator (Keyu, Dezhou, China), with 100 eggs per block (10 eggs/row × 10 rows). At 17.5 days of incubation, two rows (20 eggs) from each tier randomly received one of five inoculation treatments into the amniotic cavity, including non-injected control, 200 μL of PBS diluent (PBS control), low-dose (0.5 mg/egg), medium-dose (5 mg/egg), or high-dose MOS (10 mg/egg). A non-injected control group was included for hatchability evaluation. In the current trial, hatchability and birth weight did not differ between groups; therefore, only the injected chicks were kept for growth evaluation. To avoid potential sex effects, approximately 254 male chicks (overall hatchability 94.0%) from the inoculation treatments were placed in 28 battery cages after hatch, with up to 10 chicks per cage. Male chicks from two adjacent rows on the same tier were transferred to the same cage. In this study, the incubator tier and cage location served as environmental block factors. Chicks had ad libitum access to water and a commercial crumble feed until 21 dph. The nutrients were analyzed, and apparent metabolizable energy was calculated (Table 1).

### 2.2. In Ovo Injection Method

The in ovo injection procedure was performed according to Uni et al. [20]. Briefly, at 17.5 days of incubation, eggs were transferred to a biological safety cabinet at 37 °C. The blunt end of each egg was disinfected with 70% ethanol, and a hole (approximately 1 mm in diameter) was drilled using a sterile awl. A total of 200 μL of solution was injected into the amnion via a disposable sterile 22-gauge needle. Inoculation treatments included sterile PBS diluent and MOS at a working density of 2.5, 25, and 50 mg/mL. The working solution was prepared by mixing MOS powder (Solarbio, Beijing, China) with sterile PBS, exposing under UV for 12 h, and filtering through a 0.22 μm polyethersulfone membrane (Millex^™^-MP, Merck, Darmstadt, Germany). The holes were sealed with sterile paraffin, and the eggs were immediately returned to the incubator after cooling.

### 2.3. Growth Performance Measurements

Feed addition, residual feed, and body weight per cage were recorded on 0, 7, 14, and 21 dph. Average daily gain (ADG), average daily feed intake (ADFI), and feed conversion ratio (FCR) were calculated accordingly. Daily mortality was recorded to correct ADFI.

### 2.4. Sampling, Intestinal Morphology, and Serum Measurements

On 7, 14, and 21 dph, one bird per cage was randomly selected for cecal content collection. Three-centimeter sections of the duodenum, jejunum, and ileum were collected, gently rinsed with PBS, and fixed in formalin solution. Cecal slurry from both pouches was mixed, and approximately one gram of content per bird was collected into 2 mL cryo vials, snap-frozen in liquid nitrogen, and stored at −80 °C. On day 7, blood samples were collected following cervical dislocation. Serum samples were obtained after 4 h of clotting and centrifugation at 1200× *g* for 10 min.

Intestinal samples were labeled in a double-blind manner, and histological slides were stained with Alcian blue and periodic acid–Schiff by a commercial laboratory (Chengdu Lilai Biotechnology, Chengdu, China). Histological images were collected under 40× magnification using a biological microscope (Model: CX23, Olympus, Tokyo, Japan). Villus height, villus width, crypt depth, and muscle thickness were measured using ImageJ software (ver 1.54s, NIH), and five intact fields of view were examined for each measurement. The villus height to crypt depth ratio was calculated.

Concentrations of total protein, albumin, triglyceride, low-density lipoprotein, cholesterol, high-density lipoprotein, and glucose in serum were determined using commercial assay kits (Nanjing Jiancheng Bioengineering Institute, Nanjing, China) with a microplate reader (Model: SpectraMax iD3, Molecular Devices LLC, San Joe, CA, USA). Serum IgA levels were measured by ElaBoX^TM^ Chicken IgA ELISA Kit (Solarbio, Beijing, China).

### 2.5. Cecal Microbiota

Cecal microbiota was evaluated through 16S rRNA amplicon sequencing at a commercial laboratory (MajorBio, Shanghai, China). Briefly, genomic DNA was collected via the QIAamp Fast DNA Stool Mini kit (Qiagen, Germantown, MD, USA), and the V3–V4 region of microbial 16S rRNA was amplified using the 338 F forward primer (5′ACTCCTACGGGAGGCAGCAAG) and 806 R reverse primer (5′GGACTACHVGGGTWTCTAAT). Identifiable indexes were added through a second run of PCR, and raw sequences were generated on an Illumina HiSeq platform (Illumina, Shanghai China). Raw reads were quality-controlled using FastQC (v0.12.1). Low-quality reads were filtered out, including those with a length shorter than 110 nt, primer mismatches, or where more than 7% of bases had a Phred quality score below 20. The DADA2 plugin within QIIME 2 was utilized for denoising the high-quality sequences, which involved length filtering (minimum 420 bp), chimera removal, and dereplication to generate amplicon sequence variants (ASVs). Clean reads are available at https://www.ncbi.nlm.nih.gov/sra/PRJNA1480554 (available on 22 June 2026). Cecal microbial diversity and composition were analyzed via the Majorbio Cloud Platform (MajorBio, Shanghai, China). Genus-level alpha diversity indices, including Sobs, Shannon, and Simpson, were calculated via Mothur software (version 1.30.2), and Bray–Curtis distance matrices were calculated via QIIME software (version 1.9.1), followed by principal coordinate analysis (PCoA). Linear discriminant analysis (LDA) was performed to differentiate bacterial taxa, with criteria of LDA score > 3.5 and *p*-value < 0.050 under all-against-all multiple testing.

### 2.6. Data Analysis

The effects of MOS injection on growth performance, intestinal morphology, serum biochemicals, and microbial diversity and relative abundance levels were analyzed following the statistical model: *Yij* = *μ* + *αi* + *βj* + *εij*, where *Yij* represents the above dependent variables, *μ* denoting population means, *αi* denoting injection treatment (fixed factor), *βj* denoting block factor (random factor), and *εij* denoting random error. The incubator tier location or cage location was considered as the block factor. For bird performance data (hatchability, ADG, ADFI, and FCR), a set of eggs in the incubator (20 eggs) or a cage of chicks (up to 10) was considered the experimental unit. For histological measurements and microbial diversity and relative abundance, the sampled chick from each cage was considered the experimental unit. The ANOVA with orthogonal polynomial contrast trend analysis was performed using the GLM procedure in SAS 9.4 software (SAS Institute, Cary, NC, USA). The orthogonal polynomial coefficients of unequally spaced dosages were generated by the IML procedure in SAS 9.4. When injection treatment resulted in differences, means were compared using Duncan’s test. A non-parametric Spearman correlation analysis was conducted to reveal networks between chick performance and differential bacterial taxa. Differences were considered significant at *α* = 0.050.

## 3. Results

### 3.1. Effects of In Ovo Injection of MOS on Broiler Growth Performance

The hatchability in the non-injected control, PBS, low, medium, and high MOS injection treatments reached 94.8%, 95.3%, 92.5%, 93.8%, and 94.2%, respectively, with no statistical differences (*p* = 0.355). Growth performance from 0 to 21 dph is presented in Table 2. During the first week (0–7 dph), MOS injection linearly increased ADFI and ADG (*p* = 0.007 and 0.009); chicks that received 10 mg MOS exhibited a higher ADFI and subsequently a higher ADG than the chicks that received PBS diluent control. In addition, MOS injection quadratically affected the ADFI during 8–14 dph (*p* = 0.046), with the 0.5 mg/egg reaching the highest ADFI. However, no injection effects were found in the later growth performance during 15–21 dph or the whole-period growth performance during 0–21 dph (*p* > 0.05).

### 3.2. Effects of In Ovo Injection of MOS on Intestinal Morphology

The effects of in ovo administration of MOS on the intestinal morphology of broilers are detailed in Table 3. At 7 dph, MOS injection linearly increased duodenal villus height, villus width, and villus height-to-crypt depth ratio (*p* = 0.001, 0.006, and 0.005). Typically, birds that received 10 mg MOS demonstrated longer and wider villi and exhibited a higher villus height-to-crypt depth ratio compared to birds that received PBS control. As shown in Supplementary Figure S1, the histological examination revealed that 10 mg MOS increased villus height and width compared to the PBS control. Additionally, the duodenal muscle thickness on 7 dph was enhanced by injecting 0.5 mg MOS (*p* = 0.013). Also, no intestinal morphological improvements due to MOS injection were observed at any other age (*p* > 0.05).

### 3.3. Effects of In Ovo Injection of MOS on Serum Biochemical Parameters

Table 4 outlines serum biochemicals of chicks at 7 dph. Concentrations of albumin, protein, glucose, cholesterol, high-density lipoprotein cholesterol, low-density lipoprotein cholesterol, and immunoglobulin A were not affected by in ovo MOS treatments (all *p* > 0.05). In ovo injection of 5 mg MOS reduced serum triglyceride levels as compared to the PBS control (*p* = 0.044).

### 3.4. Effects of In Ovo Injection of MOS on Cecal Microbiota Diversity

**Figure 1:**
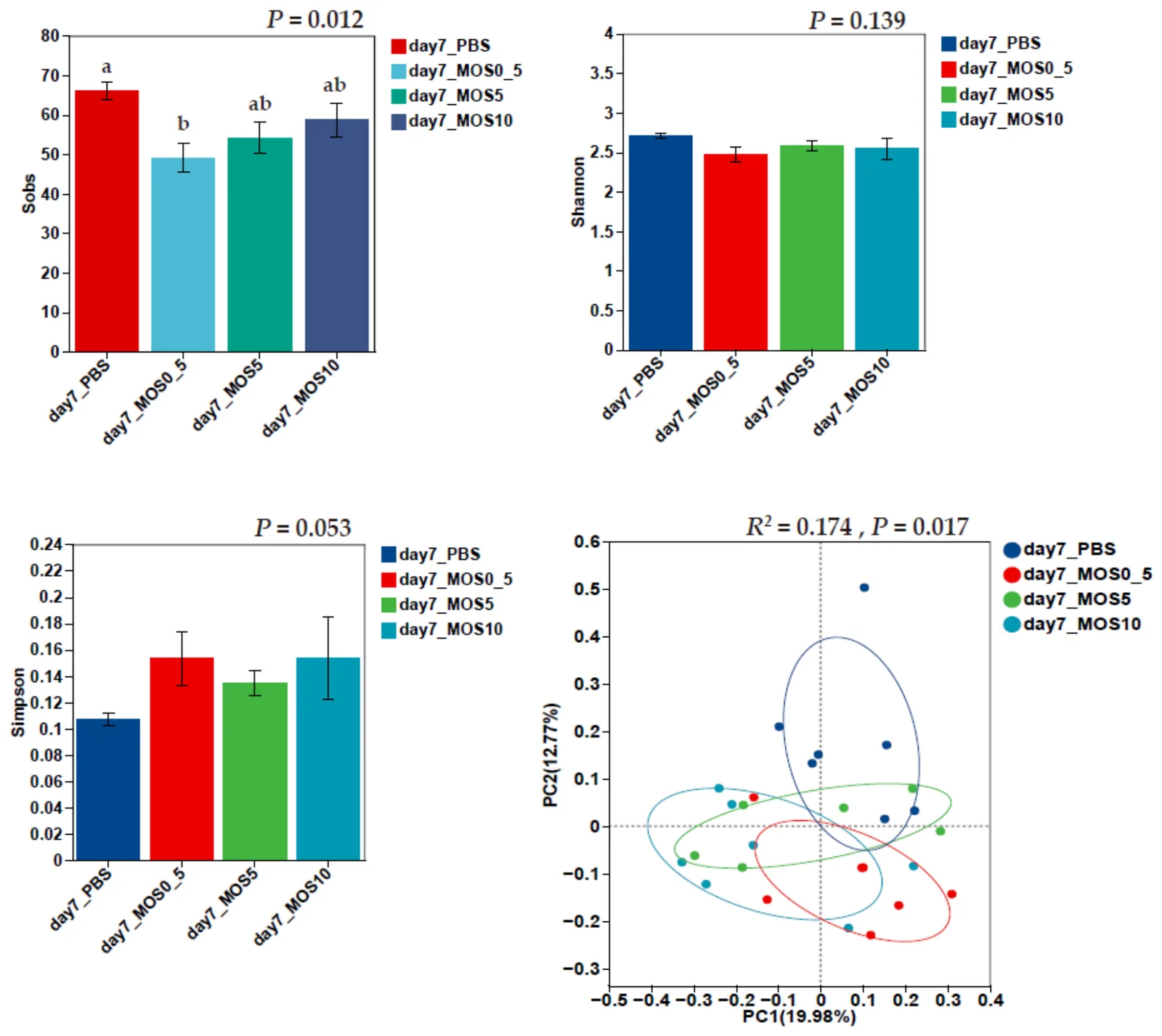
The effects of in ovo injection of MOS on cecal microbial alpha and beta diversity at 7 dph (*n* = 7). Alpha diversity indices (Sobs, Shannon, and Simpson) were analyzed by ANOVA with α = 0.050. Beta diversity was analyzed using principal coordinate analysis (PCoA) based on Bray–Curtis dissimilarity metrics and confirmed with PERMANOVA analysis. a,b: The boxes not sharing a common subscript are considered different at α = 0.050.

**Figure 2:**
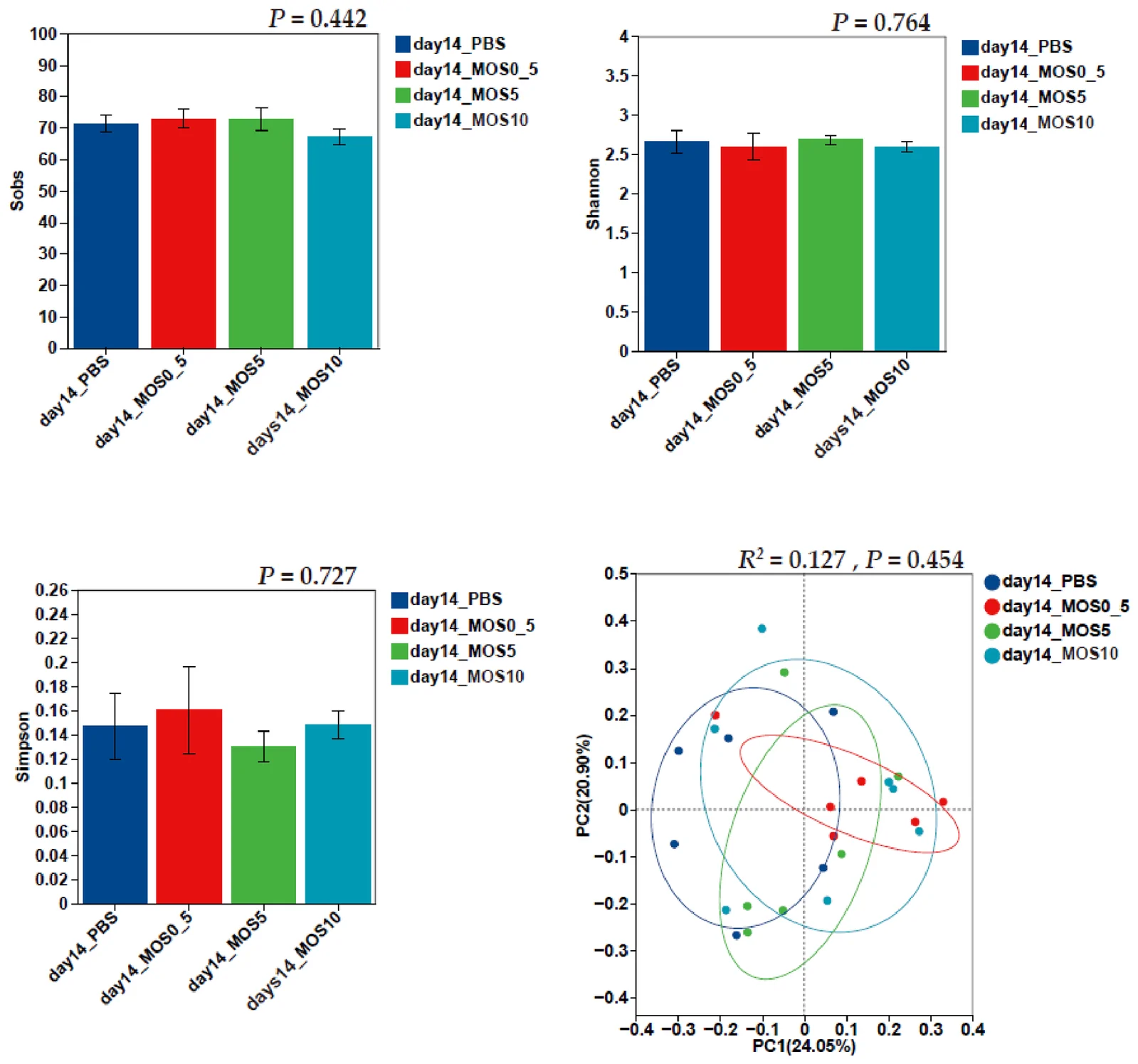
The effects of in ovo injection of MOS on cecal microbial alpha and beta diversity at 14 dph (*n* = 7). Alpha diversity indices (Sobs, Shannon, and Simpson) were analyzed by ANOVA with α = 0.050. Beta diversity was analyzed using principal coordinate analysis (PCoA) based on Bray–Curtis dissimilarity metrics and confirmed with PERMANOVA analysis.

**Figure 3:**
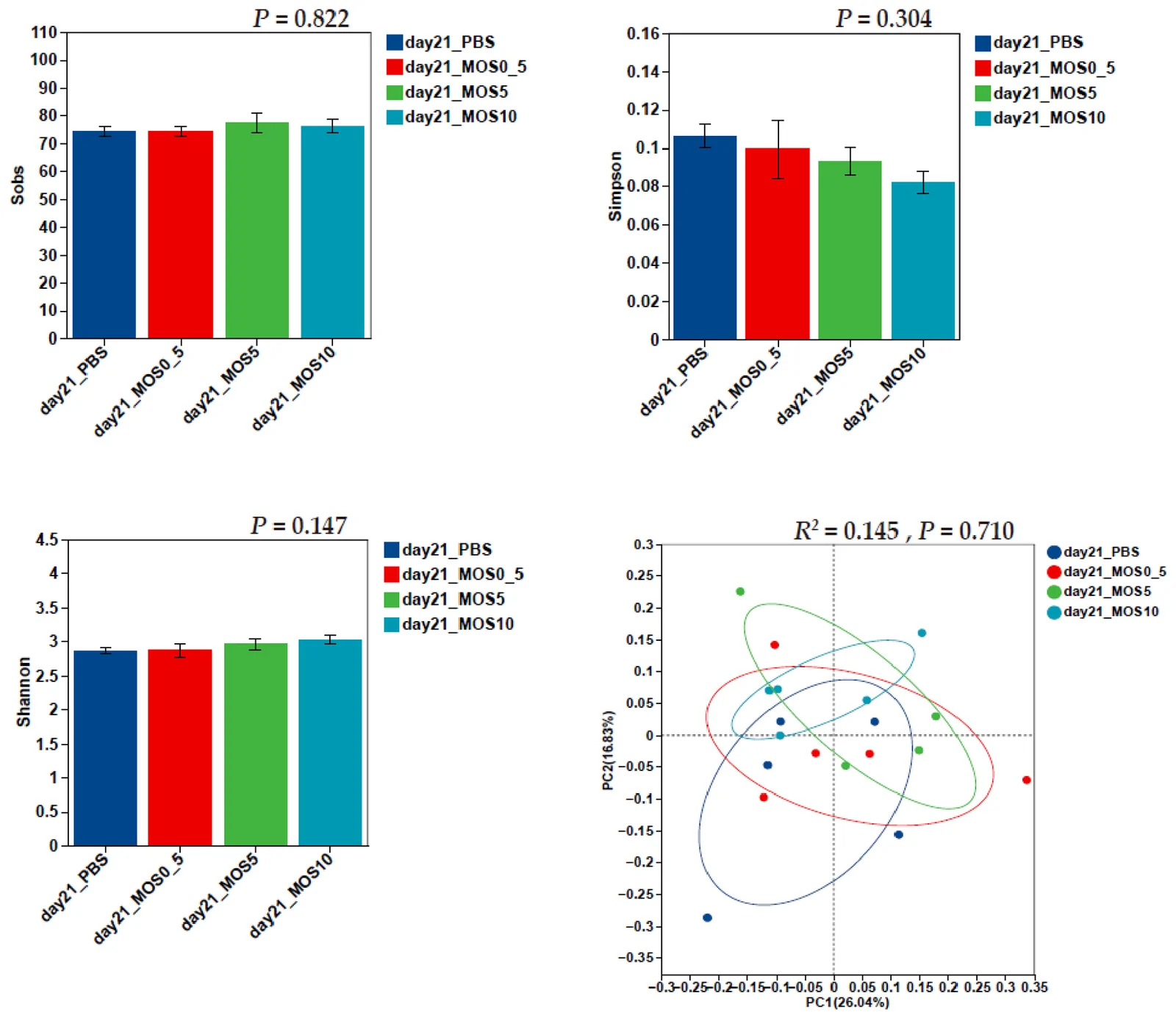
The effects of in ovo injection of MOS on cecal microbial alpha and beta diversity at 21 dph (*n* = 7). Alpha diversity indices (Sobs, Shannon, and Simpson) were analyzed by ANOVA with α = 0.050. Beta diversity was analyzed using principal coordinate analysis (PCoA) based on Bray–Curtis dissimilarity metrics and confirmed with PERMANOVA analysis.

The alpha and beta diversity of the cecal microbiota on 7, 14, and 21 dph are presented in Figure 1, Figure 2 and Figure 3. At day 7, injecting 0.5 MOS reduced the Sobs index (community richness) and showed a trend toward increasing the Simpson index compared to the PBS control (*p* = 0.012 and 0.053). Moreover, PCoA coupled with Adonis analyses (*p* = 0.017) revealed a distinct separation in community structure (beta diversity).

The in ovo injection of MOS only modulated cecal microbiota at 7 dph (Figure 1) without affecting any microbial diversity or distribution at 14 (Figure 2) or 21 dph (Figure 3).

### 3.5. Effects of In Ovo Injection of MOS on Cecal Microbial Composition

**Figure 4:**
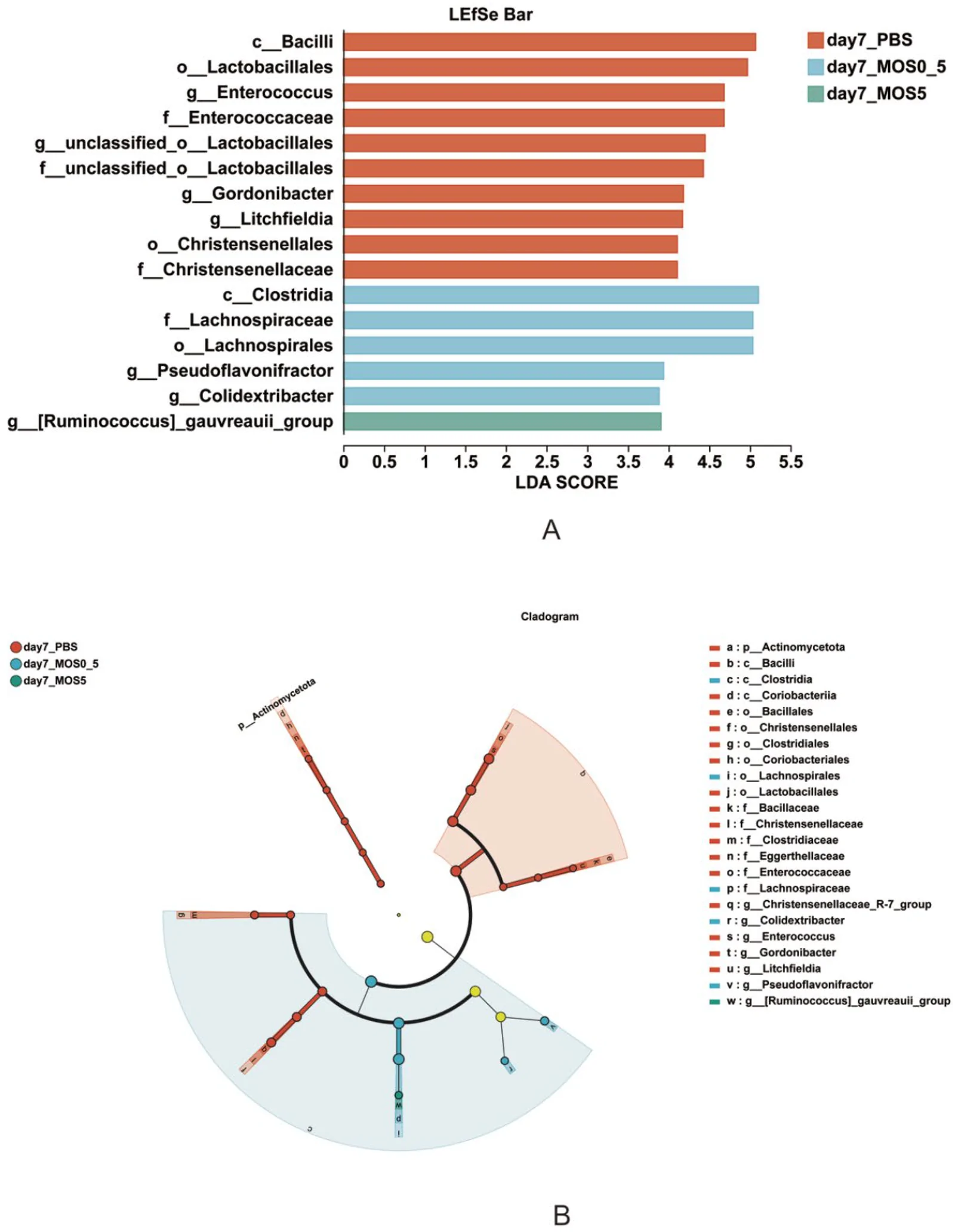
The LefSe analysis (**A**) of cecal microbiota at 7 dph using LDA scores of 3.5 or greater and a *p*-value of multiple testing less than 0.050, and the cladogram (**B**). Different-colored regions represent different groups. Circles indicate phylogenetic levels from phylum to genus. The diameter of each circle is proportional to the abundance of that group.

The LEfSe analysis with LDA greater than 3.5 and a *p*-value of multiple testing smaller than 0.050 was conducted to differentiate taxa between treatments (Figure 4). The chicks receiving PBS control were enriched in class Bacilli and order Lactobacillales, specifically genera Enterococcus, Gordonibacter, and Litchfieldia, while the MOS 0.5 mg group showed higher abundances of class Clostridia, family Lachnospiraceae, and genera Pseudoflavonifractor and Colidextribacter. The MOS5 group was enriched in the genus Ruminococcus_gauvreauii_group.

**Figure 5:**
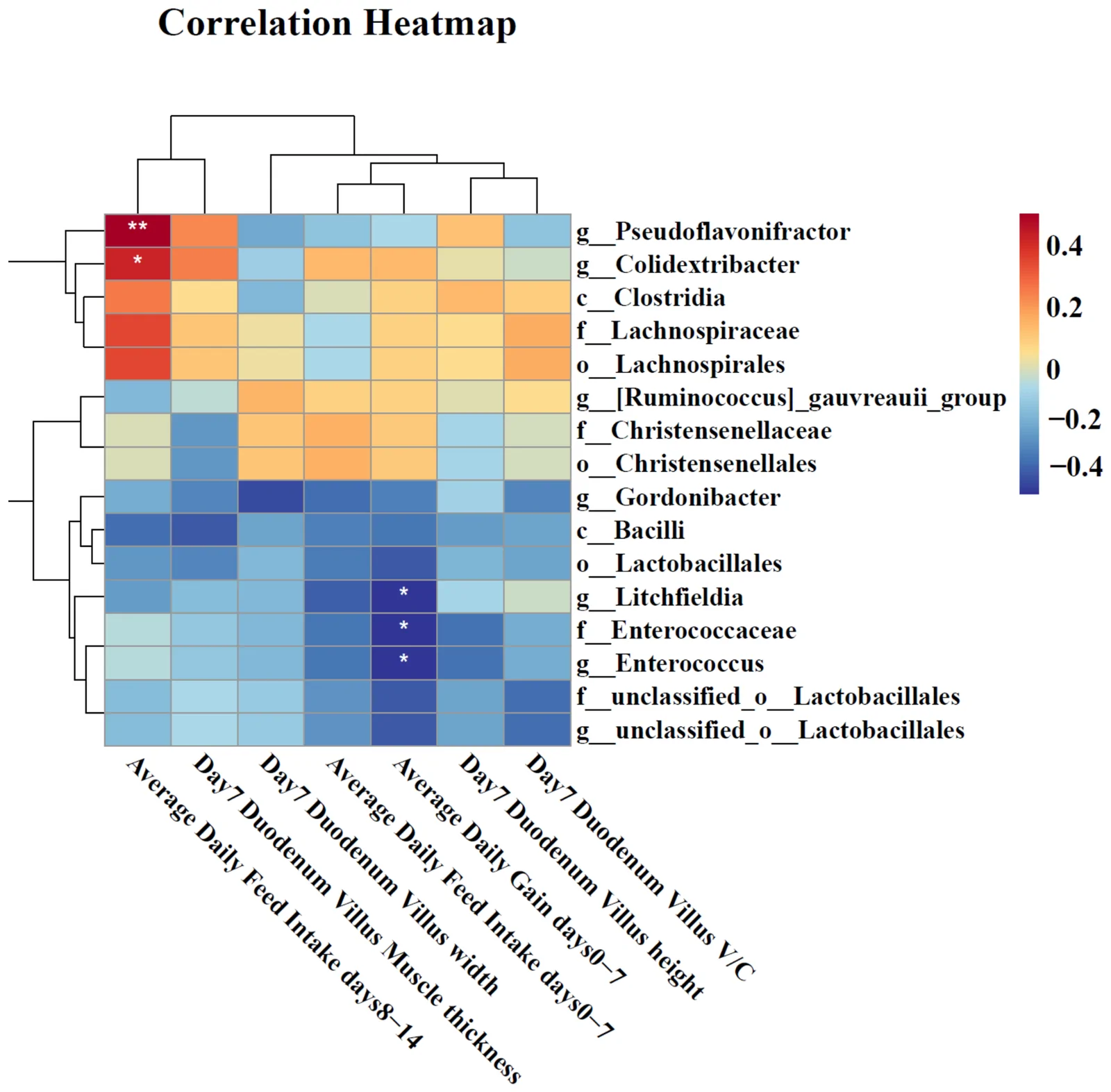
The Spearman correlation analysis between differential microbial taxa and chick performance measurements. The color intensity indicates the Spearman correlation coefficient (r), where red indicates a positive correlation, and blue indicates a negative correlation. Significance is denoted by asterisks (* *p* < 0.05, ** *p* < 0.01).

A Spearman non-parametric correlation heatmap is presented in Figure 5. The analysis revealed that enriched genera Pseudoflavonifractor and Colidextribacter in the MOS group positively correlated with ADFI from 8 to 14 dph (*p* < 0.010 and 0.050, respectively). Conversely, PBS-enriched Enterococcaceae, Enterococcus, and Litchfieldia negatively correlated with ADG from 0 to 7 dph (*p* < 0.05).

## 4. Discussion

Dietary MOS supplementation is well-established as an alternative to antibiotic growth promoters in broiler production, with reported benefits for feed intake, body weight gain, and FCR [21]. The present study demonstrates that in ovo MOS administration into the amnion promotes chick growth during the first week post-hatch, with the high dosage (10 mg/egg) specifically increasing feed consumption and weight gain. Amnion injection allows MOS to reach the developing intestine before hatch, where it serves as a fermentation substrate for the nascent microbiota and a source of SCFAs for host chicks. Compared with mannan polysaccharides, oligosaccharides exhibit faster fermentation kinetics and greater SCFA production [22]. However, the intestinal SCFA levels were not verified in the current trial. The growth-promoting efficacy of in ovo MOS, however, appears to depend on dosage, timing, and the chick’s health status. Bełdowska et al. [23] and Singh et al. [24] reported that the degree of polymerization, mannotriose (3 units) or mannotetrose (4 units), did not influence efficacy. In contrast, Dankowiakowska et al. [25] found that MOS dosage ranging from 0.3 to 5 mg/egg administered at 12 or 17.5 days of incubation failed to improve post-hatch growth performance. Notably, the growth benefit observed in the current trial was transient, with no effects persisting beyond 14 dph. Overall ADG, ADFI, or FCR over the full 0–21 dph were not affected by in ovo MOS under the current experimental condition. During the perinatal period, chicks transition from yolk-derived nutrition to exogenous feed, and microbial taxa initially favored by in ovo prebiotic administration may subsequently be displaced by environmental bacteria as yolk reserves are depleted [26].

Consistent with previous reports, in ovo administration of 10 mg MOS/egg improved intestinal architecture, as evidenced by increased villus height and villus-to-crypt ratios. Cheled-Shoval et al. [12] demonstrated that in ovo injection of 50 mg/egg at 17.5 days of incubation increased duodenal villus area and first-week BW gain. In agreement, enhanced villus height and width in MOS-treated chicks were observed in the current trial, suggesting an expanded absorptive surface area which might be associated with efficient nutrient utilization, although this may also reflect the larger body size of treated birds. Butyrate, one of the primary SCFAs that could be produced from MOS fermentation [27], was reported to promote epithelial cell self-renewal and differentiation via the Wnt/β-catenin and Notch signaling pathways [28]. Uni et al. (1998) reported that a higher villus-to-crypt ratio indicated higher enterocyte turnover [29]. Within the first 10 dph, enterocyte migration rates are consistently higher in the duodenum than in the jejunum or ileum [30], which may explain the duodenum’s heightened responsiveness to a single pre-hatch MOS injection in the current trial. After hatch, the first week represents a critical period of rapid intestinal epithelial turnover and maturation of the mucus barrier and innate immune system [31]. Interventions acting within this developmental window may confer transient morphological benefits [32]; however, as host intestinal maturation progresses and epithelial turnover dynamics change, these early advantages may gradually diminish and converge with normal developmental trajectories [33]. Nevertheless, in the absence of continued MOS supply, these structural improvements were not sustained at later ages.

Under the unchallenged conditions of this trial, in ovo MOS did not significantly affect most serum biochemical parameters, including IgA. However, serum triglyceride levels were quadratically decreased by MOS dosage, with 5 mg/egg reducing levels by 27%. This observation aligns with previous dietary supplementation studies reporting a tendency for MOS to lower serum triglycerides in broilers [34] and quails [35]. Mechanistically, dietary MOS combined with probiotic *Bifidobacterium* or *Lactobacillus* downregulates hepatic lipogenic genes (acetyl-CoA carboxylase and fatty acid synthase) while upregulating lipolytic genes (AMP-activated protein kinase and stearoyl-CoA desaturase-1), resulting in reduced body fat [36]. In intestinal epithelial cells, SCFAs upregulate the expression of glucagon-like peptide-1, which suppresses hepatic lipid accumulation via AMPK/acetyl CoA carboxylase signaling [36]. In the present study, the in ovo MOS might similarly promote SCFA-producing microbes, contributing to the observed triglyceride reduction.

Cecal microbiota analysis revealed that in ovo MOS reduced alpha diversity and modified microbial community structure at 7 dph. The cecal microbiota of newly hatched chicks undergoes rapid ecological succession, typically reaching full diversity by the second or third week post-hatch [18,37]. Nutritional interventions administered in ovo or early post-hatch can redirect this assembly process. Whereas previous studies reported no significant differences in Shannon or Chao1 diversity indices following in ovo xylo-oligosaccharide or MOS administration, or even increased diversity at 21 or 28 dph [24,38], our findings suggest that diversity changes are time-dependent. At 7 dph, MOS injection treatment reduced overall microbial diversity by enriching the abundance of obligate anaerobes. This early intestinal programming may alter the microbial community trajectory before the microbiota stabilizes. However, as with the structural improvements, the microbiota modulation was transient; by 14 dph, alpha and beta diversity did not differ between MOS-treated and control birds.

At 7 dph, PBS-injected control chicks were enriched in *Enterococcus* and *Litchfieldia*, facultative anaerobes associated with lower weight gain; whereas MOS-treated chicks were enriched in Pseudoflavonifractor and Colidextribacter, taxa linked to improved feed consumption. This compositional shift was associated with a greater relative representation of strictly anaerobic and fermentative taxa in the early cecal community following in ovo MOS administration. While Enterococcus can act as both a commensal and an opportunistic pathogen in poultry [39,40], its stable presence in early life can be reduced by butyrate-producing additives [40]. Although PBS-injected chicks exhibited lower body weight compared to MOS-treated birds, the concurrent enrichment of Enterococcus and Litchfieldia in the PBS group should not be interpreted as evidence of a growth-suppressive effect of these taxa. Given the correlational nature of the microbiota data and the multifactorial determinants of early growth, this association may simply reflect treatment-related physiological changes rather than a direct causal relationship.

The MOS-treated chicks harbored increased abundances of Pseudoflavonifractor and Colidextribacter, both obligate anaerobes and butyric acid producers. Pseudoflavonifractor is a stable chicken cecal commensal that is enriched by probiotics such as Lactobacillus plantarum GX17 [41] and in antibiotic-free rearing systems [42]. The Pseudoflavonifractor abundance has been linked to reduced inflammation and improved antioxidant capacity in lipopolysaccharide-challenged birds [43]. Similarly, Colidextribacter is considered a protective gut microbe, with increased abundance observed in *Eimeria*-resistant chicks [44]. Both genera are capable of utilizing MOS to produce butyrate, which supports enterocyte proliferation and differentiation. These microbial shifts were consistent with the improved villus morphology, feed intake, and weight gain observed in MOS-treated chicks in the current study. However, the potential contribution of the gut microbiota to host growth warrants further investigation, including characterization of microbial metabolites such as SCFAs and bioactive peptides.

Notably, by 14 and 21 dph, the cecal microbiota had stabilized across treatment groups, with no lasting differences between MOS-treated and control birds. After hatch, chicks are continuously exposed to a diverse and abundant pool of environmental microorganisms, and these environmental inputs may increasingly shape the gut microbial community during the first 1–2 weeks of life [45], potentially limiting the persistence of microbial advantages established by a single in ovo intervention. Meijerink et al. found that, regardless of whether chicks received a hen-derived microbial inoculum on the day of hatch, the microbial communities converged toward a common “mature” profile by 14 days of age [46]. Similarly, Baldwin et al. reported that the chicken gut microbiota approaches a quasi-stable state within the first week of life, after which the mature community becomes increasingly resistant to further remodeling [47]. Thus, the microbial and morphological differences observed at 7 days following in ovo administration may reflect the intervention acting during the final window before microbial community convergence. As the gut microbiota matures around 14 days of age, the early advantages conferred by a single prebiotic dosage may subsequently be attenuated through deterministic microbial succession. However, the growth-promoting and microbiota-modulating effects of this single pre-hatch intervention are transient, diminishing by 14 days post-hatch as environmental colonization and dietary transitions reshape the gut ecosystem. Our findings underscore the critical window of perinatal microbial programming. However, sustained performance benefits under commercial settings might require repeated MOS administration, whether via additional in ovo interventions or post-hatch supplementation, depending on economic and practical constraints.

## 5. Conclusions

In conclusion, in ovo administration of MOS at 10 mg/egg transiently improves growth and intestinal morphology, and MOS at 0.5 mg/egg altered cecal microbiota composition during the first week post-hatch, suggesting a potential role for MOS in shaping early microbial establishment in broilers. However, these effects dissipate by 14 dph as the microbiota converges toward a mature community.

## Figures and Tables

**Table 1:** Composition and nutrient levels of basal diets (air-dry basis, % except for energy).

Items	0–21 d
Corn	51.26
Soybean meal	41.34
Soybean oil	1.99
CaHPO_4_	2.43
Limestone	1.30
NaCl	0.33
DL-Methionine	0.32
Choline chloride	0.19
L-Threonine	0.13
L-Lysine HCl	0.11
Premix ^1^	0.60
Total	100
Nutrient levels ^2^	
Apparent Metabolizable Energy/(kcal/lb)	1412.60
Crude protein	23.09
Calcium	1.10
Total Phosphorus	0.81
Lysine	1.26
Methionine + Cystine	0.94
Threonine	0.86
Methionine	0.63

^1^ The premix provided the following nutrients per kg of diet VA 19,000 IU, VD_3_ 4000 IU, VE 24 IU, VK_3_ 2.0 mg, VB_1_ 2.6 mg, VB_2_ 1.12 mg, VB_6_ 5.6 mg, VB_12_ 0.02 mg, Mn 110 mg, Zn 100 mg, Fe 80 mg, Cu 10 mg, I 0.6 mg, Se 0.35 mg. ^2^ AME was a calculated value, whereas the other nutrients were analyzed.

**Table 2:** Effects of in ovo injection of MOS on growth performance of broilers from 0 to 21 dph (g, except for FCR).

Duration	Measurements ^2^	PBS ^1^	MOS0.5	MOS5	MOS10	SEM	*p*-Value	Linear	Quadratic
0	Birth BW	47.00	47.06	46.55	47.05	0.553	0.897	0.929	0.468
21	Final BW	910.58	963.10	910.90	957.36	47.852	0.782	0.775	0.635
	ADFI	21.42 ^b^	22.62 ^b^	22.87 ^ab^	24.50 ^a^	0.546	0.008	0.007	0.391
0–7	ADG	18.01 ^b^	19.63 ^ab^	19.64 ^ab^	21.24 ^a^	0.641	0.019	0.009	0.462
	FCR	1.19	1.16	1.17	1.15	0.021	0.656	0.241	0.757
	ADFI	56.67 ^b^	64.91 ^a^	53.99 ^b^	59.30 ^ab^	2.233	0.032	0.029	0.046
8–14	ADG	43.68	48.42	43.93	44.71	1.898	0.315	0.180	0.488
	FCR	1.31	1.34	1.24	1.33	0.046	0.529	0.470	0.193
	ADFI	88.70	94.49	90.57	77.87	5.114	0.152	0.701	0.893
15–21	ADG	61.87	63.81	59.62	64.10	7.514	0.978	0.778	0.743
	FCR	1.42	1.52	1.34	1.24	0.112	0.272	0.881	0.529
	ADFI	55.59	60.99	55.80	54.13	2.294	0.188	0.413	0.576
0–21	ADG	41.52	44.04	41.20	43.76	2.636	0.835	0.425	0.655
	FCR	1.22	1.40	1.34	1.25	0.061	0.274	0.597	0.671

^1^ PBS = phosphate-buffered saline control group (200 μL/egg), MOS0.5 = low-dose group (0.5 mg/egg), MOS5 = medium-dose group (5 mg/egg), and MOS10 = high-dose group (10 mg/egg), and *n* = 7 groups/treatment. ^2^ BW = body weight, ADFI = average daily feed intake, ADG = average daily gain, and FCR = feed conversion rate. ^a,b^ Means within a row with different superscript letters differ at α = 0.050.

**Table 3:** Effects of in ovo injection of MOS on intestinal morphology of broilers at 0 to 21 dph (mm, except for V/C).

Duration	Section	Measurements	PBS ^1^	MOS0.5	MOS5	MOS10	SEM	*p*-Value	Linear	Quadratic
		Villus height	0.776 ^b^	0.791 ^b^	0.778 ^b^	1.084 ^a^	0.060	0.005	0.001	0.045
		Villus width	0.120 ^b^	0.117 ^b^	0.143 ^ab^	0.177 ^a^	0.016	0.045	0.006	0.816
	Duodenum	Crypt depth	0.179	0.174	0.155	0.161	0.023	0.865	0.506	0.589
		V/C^2^	4.93 ^b^	4.91 ^b^	6.34 ^ab^	6.61 ^a^	0.475	0.031	0.005	0.335
		Muscle thickness	0.099 ^b^	0.131 ^a^	0.105 ^b^	0.119 ^ab^	0.007	0.013	0.256	0.157
		Villus height	0.615	0.532	0.654	0.594	0.051	0.547	0.294	0.979
		Villus width	0.135	0.138	0.172	0.178	0.013	0.078	0.021	0.249
D 7	Jejunum	Crypt depth	0.173	0.178	0.181	0.166	0.020	0.955	0.961	0.735
		V/C	3.67	3.16	3.84	3.84	0.420	0.637	0.393	0.774
		Muscle thickness	0.107	0.114	0.111	0.125	0.011	0.599	0.901	0.218
		Villus height	0.322	0.281	0.273	0.425	0.046	0.094	0.797	0.017
		Villus width	0.159	0.127	0.120	0.164	0.021	0.488	0.485	0.198
	Ileum	Crypt depth	0.153	0.129	0.146	0.155	0.013	0.441	0.691	0.427
		V/C	2.20	2.13	1.95	2.92	0.376	0.275	0.883	0.065
		Muscle thickness	0.135	0.109	0.123	0.156	0.019	0.287	0.597	0.390
		Villus height	0.851	1.048	0.964	0.873	0.073	0.278	0.877	0.458
		Villus width	0.168	0.155	0.162	0.133	0.009	0.094	0.911	0.017
	Duodenum	Crypt depth	0.146	0.182	0.167	0.172	0.016	0.434	0.838	0.601
		V/C	6.25	6.34	6.47	5.67	0.661	0.818	0.945	0.372
		Muscle thickness	0.146	0.145	0.125	0.145	0.015	0.662	0.270	0.560
		Villus height	0.643	0.717	0.600	0.589	0.060	0.366	0.212	0.686
		Villus width	0.150	0.183	0.185	0.145	0.018	0.257	0.497	0.203
D 14	Jejunum	Crypt depth	0.205	0.200	0.180	0.184	0.020	0.720	0.398	0.513
		V/C	3.28	4.20	3.57	3.32	0.580	0.672	0.791	0.747
		Muscle thickness	0.134	0.130	0.122	0.116	0.009	0.565	0.349	0.313
		Villus height	0.373	0.357	0.364	0.304	0.030	0.302	0.849	0.068
		Villus width	0.151	0.201	0.186	0.160	0.019	0.233	0.817	0.779
	Ileum	Crypt depth	0.150	0.161	0.176	0.158	0.022	0.821	0.371	0.826
		V/C	2.58	2.44	2.40	2.09	0.326	0.728	0.654	0.319
		Muscle thickness	0.136	0.126	0.096	0.131	0.015	0.305	0.148	0.597
		Villus height	0.924	0.740	0.859	1.064	0.079	0.067	0.736	0.273
		Villus width	0.134	0.133	0.143	0.176	0.013	0.167	0.342	0.076
	Duodenum	Crypt depth	0.138	0.140	0.163	0.165	0.018	0.815	0.382	0.864
		V/C	7.20	5.38	5.78	7.06	1.214	0.621	0.799	0.591
		Muscle thickness	0.173	0.116	0.109	0.138	0.023	0.127	0.154	0.424
		Villus height	0.654	0.540	0.573	0.646	0.064	0.403	0.606	0.773
		Villus width	0.149	0.141	0.168	0.187	0.014	0.143	0.137	0.149
D 21	Jejunum	Crypt depth	0.203	0.148	0.142	0.219	0.052	0.615	0.902	0.424
		V/C	4.46	4.13	4.63	3.64	0.803	0.824	0.855	0.371
		Muscle thickness	0.125	0.113	0.112	0.108	0.010	0.518	0.558	0.220
		Villus height	0.370	0.333	0.334	0.371	0.024	0.474	0.461	0.695
		Villus width	0.146	0.138	0.129	0.188	0.010	0.310	0.663	0.300
	Ileum	Crypt depth	0.120	0.168	0.141	0.122	0.025	0.383	0.938	0.741
		V/C	3.46	2.36	2.73	3.23	0.419	0.278	0.779	0.601
		Muscle thickness	0.123	0.129	0.114	0.115	0.009	0.444	0.358	0.230

^1^ PBS = phosphate-buffered saline control group (200 μL/egg), MOS0.5 = low-dose group (0.5 mg/egg), MOS5 = medium-dose group (5 mg/egg), MOS10 = high-dose group (10 mg/egg), and *n* = 7 chicks/treatment. ^2^ V/C = villus height to crypt depth ratio. ^a,b^ Means within a row with different superscript letters differ at α = 0.050.

**Table 4:** The effects of in ovo injection of MOS on serum biochemicals of broilers at 7 dph.

Measurements	PBS ^1^	MOS0.5	MOS5	MOS10	SEM	*p*-Value	Linear	Quadratic
Albumin, g/L	12.52	12.93	12.31	12.24	0.763	0.908	0.890	0.722
Total protein, g/L	31.91	34.43	33.56	33.95	1.235	0.533	0.165	0.776
Glucose, mmol/L	16.82	17.11	17.56	17.68	1.190	0.951	0.751	0.720
Triglyceride, mmol/L	2.59 ^a^	2.25 ^ab^	1.88 ^b^	2.12 ^ab^	0.157	0.044	0.124	0.015
Cholesterol, mmol/L	8.50	8.51	8.66	8.88	0.524	0.947	0.800	0.866
High-density lipoprotein cholesterol, mmol/L	2.21	1.95	1.94	2.37	0.285	0.659	0.938	0.568
Low-density lipoprotein cholesterol, mmol/L	1.28	1.93	2.11	2.11	0.398	0.479	0.217	0.318
Immunoglobulin A, g/mL	207.1	224.4	217.8	224.6	10.74	0.639	0.213	0.883

^1^ PBS = phosphate-buffered saline control group (200 μL/egg), MOS0.5 = low-dose group (0.5 mg/egg), MOS5 = medium-dose group (5 mg/egg), MOS10 = high-dose group (10 mg/egg), and *n* = 7 chicks/treatment. ^a,b^ Means within a row with different superscript letters differ at α = 0.050.

## Data Availability

The 16S rRNA sequencing data are available at the website: https://www.ncbi.nlm.nih.gov/sra/PRJNA1480554 (accessed on 22 June 2026). The growth performance and histological data are available by contacting the corresponding author at wangxi@swun.edu.cn.

## Supplementary Materials

The following supporting information can be downloaded at https://www.mdpi.com/article/10.3390/ani16172802/s1. Figure S1. Representative histological images of duodenal section stained with PAS-AB. PBS = phosphate-buffered saline control group (200 μL/egg), MOS0.5 = low-dose group (0.5 mg/egg), MOS5 = medium-dose group (5 mg/egg), MOS10 = high-dose group (10 mg/egg), and *n* = 7 chicks/treatment. Scale bars = 100 µm.

## Abbreviations

The following abbreviations are used in this manuscript:

MOS	Mannan oligosaccharides
DPH	Day post hatch
AME	Apparent metabolizable energy
ANOVA	Analysis of variance
ADFI	Average daily feed intake
ADG	Average daily gain
FCR	Feed conversion ratio
SEM	Standard error of the mean
ASV	Amplicon sequence variant
PCoA	Principal coordinate analysis
LDA	Linear discriminant analysis
LEfSe	Linear discriminant analysis effect size

## References

[1] Huang N., Ma Y., Chai J., Li Z., You X., Wang X., Huang Y., Shi H. (2024). In ovo injection dosage of Lactobacillus rhamnosus on intestinal health and microbial composition of yellow broilers with or without Eimeria challenge. J. Appl. Poult. Res..

[2] Oakley B.B., Lillehoj H.S., Kogut M.H., Kim W.K., Maurer J.J., Pedroso A., Lee M.D., Collett S.R., Johnson T.J., Cox N.A. (2014). The chicken gastrointestinal microbiome. FEMS Microbiol. Lett..

[3] Rychlik I. (2020). Composition and function of chicken gut microbiota. Animals.

[4] Ramírez G.A., Richardson E., Clark J., Keshri J., Drechsler Y., Berrang M.E., Meinersmann R.J., Cox N.A., Oakley B.B. (2020). Broiler chickens and early life programming: Microbiome transplant-induced cecal community dynamics and phenotypic effects. PLoS ONE.

[5] Pender C.M., Kim S., Potter T.D., Ritzl M.M., Young M., Dalloul R.A. (2017). In ovo supplementation of probiotics and its effects on performance and immune-related gene expression in broiler chicks. Poult. Sci..

[6] Gibson G.R., Roberfroid M.B. (1995). Dietary modulation of the human colonic microbiota: Introducing the concept of prebiotics. J. Nutr..

[7] Baurhoo B., Phillip L., Ruiz-Feria C.A. (2007). Effects of purified lignin and mannan oligosaccharides on intestinal integrity and microbial populations in the ceca and litter of broiler chickens. Poult. Sci..

[8] Dhawan S., Kaur J. (2007). Microbial mannanases: An overview of production and applications. Crit. Rev. Biotechnol..

[9] Shehata A.A., Yalçın S., Latorre J.D., Basiouni S., Attia Y.A., Abd El-Wahab A., Visscher C., El-Seedi H.R., Huber C., Hafez H.M. (2022). Probiotics, prebiotics, and phytogenic substances for optimizing gut health in poultry. Microorganisms.

[10] Park S.H., Gibson K.E., Almeida G., Ricke S.C. (2014). Assessment of gastrointestinal microflora in pasture-raised chickens fed two commercial prebiotics. J. Probiotics Health.

[11] Finucane M., Spring P., Newman K.E. (1999). Incidence of mannose sensitive adhesions in enteric bacteria. Poult. Sci..

[12] Cheled-Shoval S.L., Amit-Romach E., Barbakov M., Uni Z. (2011). The effect of in ovo administration of mannan oligosaccharide on small intestine development during the pre- and posthatch periods in chickens. Poult. Sci..

[13] Smirnov A., Tako E., Ferket P.R., Uni Z. (2006). Mucin gene expression and mucin content in the chicken intestinal goblet cells are affected by in ovo feeding of carbohydrates. Poult. Sci..

[14] Hooge D.M. (2004). Meta-analysis of broiler chicken pen trials evaluating dietary mannan oligosaccharide, 1993–2003. Int. J. Poult. Sci..

[15] Spring P., Wenk C., Dawson K.A., Newman K.E. (2000). The effects of dietary mannaoligosaccharides on cecal parameters and the concentrations of enteric bacteria in the ceca of salmonella-challenged broiler chicks. Poult. Sci..

[16] Jha R., Fouhse J.M., Tiwari U.P., Li L., Willing B.P. (2019). Dietary fiber and intestinal health of monogastric animals. Front. Vet. Sci..

[17] Kogut M.H. (2019). The effect of microbiome modulation on the intestinal health of poultry. Anim. Feed Sci. Technol..

[18] Sharma S., Seekatz A., Alizadeh M., Hassan H., Yitbarek A., Pratt S., Abdelaziz K. (2026). Early-life supplementation of poultry-derived lactobacilli drives microbial succession and gut immune modulation in broiler chickens. Sci. Rep..

[19] Ballou A.L., Ali R.A., Mendoza M.A., Ellis J.C., Hassan H.M., Croom W.J., Koci M.D. (2016). Development of the chick microbiome: How early exposure influences future microbial diversity. Front. Vet. Sci..

[20] Uni Z., Ferket P.R., Tako E., Kedar O. (2005). In ovo feeding improves energy status of late-term chicken embryos. Poult. Sci..

[21] Polidoro B.R., de Oliveira M.J.K., Braga F.D.S.C., Polycarpo G.D.V. (2025). Mannan oligosaccharide as an alternative to infeed antibiotics to improve growth performance of broilers: A meta-analysis. Br. Poult. Sci..

[22] Wang J., Ke S., Strappe P., Ning M., Zhou Z. (2023). Structurally orientated rheological and gut microbiota fermentation property of mannans polysaccharides and oligosaccharides. Foods.

[23] Bełdowska A., Siwek M., Biesek J., Barszcz M., Tuśnio A., Gawin K., Dunisławska A. (2024). Impact of in ovo administration of xylo- and mannooligosaccharides on broiler chicken gut health. Poult. Sci..

[24] Singh A.K., Tiwari U.P., Mishra B., Jha R. (2022). Effects of in ovo delivered xylo- and mannan-oligosaccharides on growth performance, intestinal immunity, cecal short-chain fatty acids, and cecal microbiota of broilers. J. Anim. Sci. Biotechnol..

[25] Dankowiakowska A., Bogucka J., Sobolewska A., Tavaniello S., Maiorano G., Bednarczyk M. (2019). Effects of in ovo injection of prebiotics and synbiotics on the productive performance and microstructural features of the superficial pectoral muscle in broiler chickens. Poult. Sci..

[26] Kpodo K.R., Proszkowiec-Weglarz M. (2023). Physiological effects of in ovo delivery of bioactive substances in broiler chickens. Front. Vet. Sci..

[27] Mookiah S., Sieo C.C., Ramasamy K., Abdullah N., Ho Y.W. (2014). Effects of dietary prebiotics, probiotic and synbiotics on performance, caecal bacterial populations and caecal fermentation concentrations of broiler chickens. J. Sci. Food Agric..

[28] Li R., Quan T., Chen Y., Gao T. (2026). Butyrate improves dextran sulfate sodium-induced imbalance of intestinal stem cell homeostasis in broilers. Poult. Sci..

[29] Uni Z., Noy Y., Sklan D. (1999). Posthatch development of small intestinal function in the poult. Poult. Sci..

[30] Geyra A., Uni Z., Sklan D. (2001). Enterocyte dynamics and mucosal development in the posthatch chick. Poult. Sci..

[31] Bar-Shira E., Friedman A. (2006). Development and adaptations of innate immunity in the gastrointestinal tract of the newly hatched chick. Dev. Comp. Immunol..

[32] Schokker D., Jansman A.J.M., Veninga G., de Bruin N., Vastenhouw S.A., de Bree F.M., Bossers A., Rebel J.M.J., Smits M.A. (2017). Perturbation of microbiota in one-day old broiler chickens with antibiotic for 24 hours negatively affects intestinal immune development. BMC Genom..

[33] Shehata A.M., Paswan V.K., Attia Y.A., Abdel-Moneim A.-M.E., Abougabal M.S., Sharaf M., Elmazoudy R., Alghafari W.T., Osman M.A., Farag M.R. (2021). Managing gut microbiota through in ovo nutrition influences early-life programming in broiler chickens. Animals.

[34] Dev K., Begum J., Biswas A., Mir N.A., Singh J., Prakash R., Sonowal J., Bharali K., Tomar S., Kant R. (2021). Hepatic transcriptome analysis reveals altered lipid metabolism and consequent health indices in chicken supplemented with dietary Bifidobacterium bifidum and mannan-oligosaccharides. Sci. Rep..

[35] Iqbal M.A., Roohi N., Khan O. (2018). Dietary supplemented effects of mannan-oligosaccharides on biochemical parameters of 4 close-bred flocks of Japanese quail breeders. Poult. Sci..

[36] Zhang J.-M., Sun Y.-S., Zhao L.-Q., Chen T.-T., Fan M.-N., Jiao H.-C., Zhao J.-P., Wang X.-J., Li F.-C., Li H.-F. (2019). SCFAs-induced GLP-1 secretion links the regulation of gut microbiome on hepatic lipogenesis in chickens. Front. Microbiol..

[37] Yin Z., Ji S., Yang J., Guo W., Li Y., Ren Z., Yang X. (2023). Cecal microbial succession and its apparent association with nutrient metabolism in broiler chickens. mSphere.

[38] Das R., Mishra P., Mishra B., Jha R. (2024). Effect of in ovo feeding of xylobiose and xylotriose on plasma immunoglobulin, cecal metabolites production, microbial ecology, and metabolic pathways in broiler chickens. J. Anim. Sci. Biotechnol..

[39] Stege P.B., Schokker D., Harders F., Kar S.K., Stockhofe N., Perricone V., Rebel J.M.J., de Jong I.C., Bossers A. (2024). Diet-induced changes in the jejunal microbiota of developing broilers reduce the abundance of Enterococcus hirae and Enterococcus faecium. BMC Genom..

[40] O’Dea F., Dupuis A., Brown J., Edwards J., Kay S., Stafford G.P., Mesnage S. (2025). Phenotypic and genomic analysis of the emerging poultry pathogen Enterococcus cecorum in UK isolates. Microb. Genom..

[41] Yin Y., Liao Y., Li J., Pei Z., Wang L., Shi Y., Peng H., Tan Y., Li C., Bai H. (2023). Lactobacillus plantarum GX17 benefits growth performance and improves functions of intestinal barrier/intestinal flora among yellow-feathered broilers. Front. Immunol..

[42] Karimi S.H., Abdelaziz K., Spahany H., Astill J., Trott D., Wang B., Wang A., Parkinson J., Sharif S. (2024). Correction: Intestinal microbiome profiles in broiler chickens raised without antibiotics exhibit altered microbiome dynamics relative to conventionally raised chickens. PLoS ONE.

[43] Kong L., Wang Z., Xiao C., Zhu Q., Song Z. (2022). Glycerol monolaurate attenuated immunological stress and intestinal mucosal injury by regulating the gut microbiota and activating AMPK/Nrf2 signaling pathway in lipopolysaccharide-challenged broilers. Anim. Nutr..

[44] Qiao Y., Feng Q., Wang Q., Zhao Q., Zhu S., Zhao F., Wang Z., Zhang R., Wang J., Yu Y. (2024). Alteration in the gut microbiota of chickens resistant to Eimeria tenella infection. Microorganisms.

[45] Torok V.A., Hughes R.J., Ophel-Keller K., Ali M., MacAlpine R. (2009). Influence of different litter materials on cecal microbiota colonization in broiler chickens. Poult. Sci..

[46] Meijerink N., Kers J.G., Velkers F.C., van Haarlem D.A., Lamot D.M., de Oliveira J.E., Smidt H., Stegeman J.A., Rutten V.P.M.G., Jansen C.A. (2020). Early life inoculation with adult-derived microbiota accelerates maturation of intestinal microbiota and enhances NK cell activation in broiler chickens. Front. Vet. Sci..

[47] Baldwin S., Hughes R.J., Hao Van T.T., Moore R.J., Stanley D. (2018). At-hatch administration of probiotic to chickens can introduce beneficial changes in gut microbiota. PLoS ONE.

