# Supplementary material for: Effects of In Ovo Injection of Mannan-Oligosaccharides on Early Growth Performance, Intestinal Morphology, and Cecal Microbiota in Broilers
Source: Animals (Basel). 2026 Sep 6;16(17):2802. doi: 10.3390/ani16172802 (PMC13564829; doi:10.3390/ani16172802)
Supplement: Supplementary file 1 [file animals-16-02802-s001.zip › animals-4498830-Supplementary Figure S1.pdf]

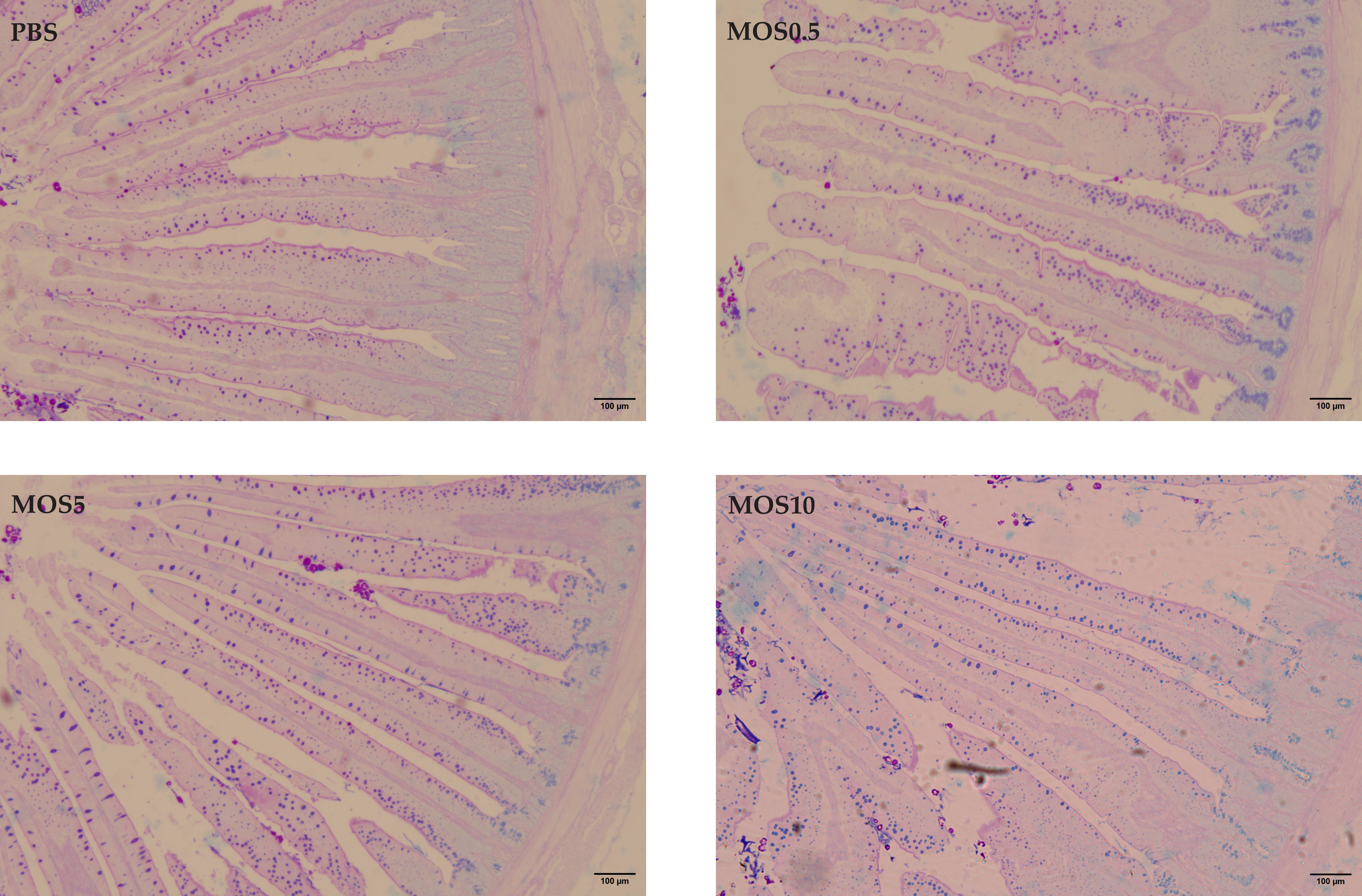
PBS
100 μm
MOS0.5
100 μm
MOS5
100 μm
MOS10
100 μm
